# Communication from J. J. Caldwell, M. D., Zebulon, Ga.

**Published:** 1871-06

**Authors:** J. J. Caldwell

**Affiliations:** Zebulon, Ga.


ZEBULON, Ga., May 9, 1871.

*Editors Atlanta Medical Journal:* Inclosed please find $5, my subscription for your valuable Journal.

In regard to the Georgia Medical Association, allow me to say, I trust that personal matters are out of it for all time to come, and that all can now go to work to advance the principles of "legitimate medicine." Let all strive for fraternal union, and the promotion of the great truths of a "time-honored profession." Then may we see empiricism banished from our midst.

At the risk of being tedious, I am inclined to give you the history of a patient, and ask your opinion on a few points connected with the case. It is one in which I have felt very great interest.

Mrs. B., æt. forty-one years, a stout, healthy woman, of nervous temperament, has been married twice. She lived with her first husband twelve or fifteen years, but bore no children by him. About one year after her second marriage in 1865, I was called in haste to see her, and found that she had been in labor forty-eight hours, attended by a *reformer*. I could get nothing satisfactory from him, further than that all went on well up to six or eight hours before I was sent for. At this time her pains had nearly ceased, general strength exhausted, with feeble circulation. I found the presentation favorable, second vertex, with the head firmly impacted in the pelvis, so much so as not to admit of any movement by pressure with the hand. I was satisfied the child had been dead some hours. Under these circumstances I determined to operate at once, and save the woman if possible. Lessening the size of the head by craneotomy, I delivered her of a large male child. From the best examination I could make under the circumstances, it was determined that the long diameter of the pelvis was about one inch under the usual size. This necessarily caused fears for her safety in the event she should again become pregnant. She convalesced slowly, but finally recovered, and again became pregnant in 1866. She was delivered the second time, after using every other means I could without success, by craneotomy. This time she recovered rapidly without untoward symptom. In 1868 she became pregnant again, and seemed very apprehensive, during her period of gestation, that this would be her last and closing labor. I could but feel a deep interest for her, and cheered her all in my power, that she might go through safely. When the time came I was summoned immediately. I had previously prepared her system as well as possible, in order that she might have every chance possible. Her labor progressed rapidly, and as soon as the os was sufficiently dilated, I gave her Ergot freely, in order to assist, or rather strengthen, the expulsive efforts of nature. She became extremely nervous and restless; begged that I would operate again. I asked her to rest satisfied; that as soon as I was convinced she could not be delivered without, I would certainly operate, and that I would not wait long enough to endanger her life by the delay; also, that I felt extremely anxious she should be delivered of a living child. In about an hour she was safely delivered of a small female child. It is now a sprightly little girl. On the 3d of May, instant, I was called to attend her in her fourth labor. It is to this one I would call your attention particularly. Her health has been poor during the time of her last pregnancy. She has suffered very severely with neuralgia of her face, left side and shoulder, for several months prior to her confinement. Some eight weeks ago I was called to see her, and found her suffering from deep-seated abscess of her left breast. It went on to suppuration and was lanced three or four times. It is still giving her trouble. Six weeks before her confinement I was called in haste to see her, and found her bleeding profusely. I think she must have lost at least five or six pints of blood. Upon examination I found the vagina filled entirely with coagula. On removing the clots, I found no dilatation of the os, and with the aid of an opiate she became quiet; the flooding ceased; some sanguineous discharge continuing for four or five days after. I enjoined perfect quiet and rest in horizontal posture for one week. Upon examining the clots (as I supposed them to be) closely, I found one about the size of a turkey's egg, seemingly more consistent than the rest, and upon immersing it in water, and cleaning it thoroughly, it was thought to be not a coagula, but had the appearance of a placenta without any uniformity of shape or the usual appearance of a true placenta. I regret very much that I did not preserve it for further examination—neglected on account of my interest for the lady at the time, when one of the lady friends, thinking there would be no further use for it, emptied it with the coagula into the fire. Thus I was deprived of any further examination of it.

Now, what was it? Was it a false conception? And is it possible for a true or genuine and false conception to exist at the same time? These are the points I desired to have your opinion on. You will understand that six weeks after this occurrence took place, on the 3d instant, I succeeded in delivering her of a small boy child at full term of utero gestation. The mother and child are both doing tolerably well at this writing. Weight of child at delivery, six pounds; weight of lady in health, one hundred and thirty pounds. Her last labor was very hard, and somewhat protracted, owing, I suppose, to her feeble health and the fact that she has a contracted pelvis.

Now, Doctor, I have given you a rough outline of my case, and wish your opinion on points above stated when you have leisure. Was I justifiable in resorting to craneotomy in her first and second labors, as I have since succeeded in delivering her of two small living children? I felt that I was, but would have your opinion also. I am satisfied she could not possibly have been delivered without the aid of instruments. I have not gone into the minutiæ of the case, as it would make my letter, already too long, irksome to you. Very truly, your friend,

J. J. Caldwell, M. D.

[To the first interrogatory in the above letter we must answer, that without expressing any opinion as to its origin, whether the result of conception in any form or not, the hemorrhage certainly proceeded from some other connection with the uterus than that of the placenta of the child, which was afterwards born at the full term of utero gestation, else in all probability it would have been expelled at the time of hemorrhage, or would have perished from detachment of the placenta sufficient to produce such a loss of blood, Might not a polypus, or some such growth upon the os or cervix uteri, unconnected with conception, account for this anomalous state of things?

As to the propriety of the first and second delivery by craneotomy, it would seem from the history given that this course was presented as the alternative for the mother's safety, and was therefore justifiable. Much as we deplore the circumstances which give rise to such necessity, censure should rather attach to him who sacrifices both mother and child, when in his power to save one. Were it perfectly certain early in labor that the head could not pass through the pelvis without mechanical interference, duty might compel a choice between the several means of Cæsarian section the forceps and craneotomy. Unfortunately for the child, however, as in the cases presented, we cannot always avail ourselves of this knowledge.]

